# Incidence of Upper Extremity Deep Vein Thrombosis in Acute Leukemia and Effect on Mortality

**DOI:** 10.1055/s-0040-1718883

**Published:** 2020-10-28

**Authors:** Christina Poh, Ann Brunson, Theresa Keegan, Ted Wun, Anjlee Mahajan

**Affiliations:** 1 Center for Oncology Hematology Outcomes Research and Training (COHORT), Division of Hematology Oncology, University of California, Davis School of Medicine, Sacramento, California, United States; 2 Division of Medical Oncology, Department of Medicine, University of Washington, Seattle, Washington, United States; 3 UC Davis Clinical and Translational Science Center, University of California, Davis, Sacramento, California, United States

**Keywords:** upper extremity deep vein thrombosis, deep vein thrombosis, venous thromboembolism, acute leukemia

## Abstract

The cumulative incidence, risk factors, rate of subsequent venous thromboembolism (VTE) and bleeding and impact on mortality of isolated upper extremity deep vein thrombosis (UE DVT) in acute leukemia are not well-described. The California Cancer Registry, used to identify treated patients with acute myeloid leukemia (AML) and acute lymphoblastic leukemia (ALL) diagnosed between 2009 and 2014, was linked with the statewide hospitalization database to determine cumulative incidences of UE DVT and subsequent VTE and bleeding after UE DVT diagnosis. Cox proportional hazards regression models were used to assess the association of UE DVT on the risk of subsequent pulmonary embolism (PE) or lower extremity deep vein thrombosis (LE DVT) and subsequent bleeding, and the impact of UE DVT on mortality. There were 5,072 patients identified: 3,252 had AML and 1,820 had ALL. Three- and 12-month cumulative incidences of UE DVT were 4.8% (95% confidence interval [CI]: 4.1–5.6) and 6.6% (95% CI: 5.8–7.5) for AML and 4.1% (95% CI: 3.2–5.1) and 5.9% (95% CI: 4.9–7.1) for ALL, respectively. Twelve-month cumulative incidences of subsequent VTE after an incident UE DVT diagnosis were 5.3% for AML and 12.2% for ALL. Twelve-month cumulative incidences of subsequent bleeding after an incident UE DVT diagnosis were 15.4% for AML and 21.1% for ALL. UE DVT was associated with an increased risk of subsequent bleeding for both AML (hazard ratio [HR]: 2.07; 95% CI: 1.60–2.68) and ALL (HR: 1.62; 95% CI: 1.02–2.57) but was not an independent risk factor for subsequent PE or LE DVT for either leukemia subtype. Isolated incident UE DVT was associated with increased leukemia-specific mortality for AML (HR: 1.42; 95% CI: 1.16–1.73) and ALL (HR: 1.80; 95% CI: 1.31–2.47). UE DVT is a relatively common complication among patients with AML and ALL and has a significant impact on bleeding and mortality. Further research is needed to determine appropriate therapy for this high-risk population.

## Introduction

Venous thromboembolism (VTE) is a known complication of cancer. It is estimated that 20 to 30% of all primary VTE events are cancer-associated and patients with malignancy are four to seven times more likely to develop VTE compared with patients without cancer. 1 2 In addition to inherent morbidity and potential to delay therapy, VTE is reported to be the second leading cause of death in cancer patients after death from the malignancy itself. 3 Thus, prevention and treatment of VTE are common and clinically relevant issues for those with malignancy.

The risk of VTE in cancer patients varies widely among different malignancies. It is generally thought that adenocarcinomas such as pancreatic and gastric cancer are associated with a much higher VTE risk than hematologic malignancies such as myeloma, lymphoma, or leukemia. 4 5 However, a population-based cohort analysis found the incidence of VTE in acute leukemia to be comparable with the incidence in many solid tumors. 6 In addition, another population-based study concluded that patients with hematologic malignancies were up to 26 times more likely to develop VTE compared with the general population. 7

Cancer-associated upper extremity deep vein thrombosis (UE DVT) involving the brachial, axillary, jugular, and superior vena cava veins is not as well-described as VTE in other sites and its impact on prognosis remains controversial. In a single registry study of all patients with VTE, UE DVT in patients with cancer was associated with an increased incidence of recurrent VTE and death compared with UE DVT in patients without cancer. 8 In addition, previous literature published in the field of distal lower extremity deep vein thrombosis (LE DVT) suggests that VTE, regardless of location, may have a significant impact on prognosis in the presence of certain risk factors such as cancer. 9 10

We had previously reported a relatively low incidence of UE DVT in patients with acute leukemia (12-month UE DVT cumulative incidences of 1.5% for acute myeloid leukemia (AML) and 0.9% for acute lymphoblastic leukemia (ALL)); however, we cautioned that these might be underestimates due to a lack of specific diagnostic codes for UE DVT at the time. 6 Therefore, with the availability of specific coding for UE DVT after October 1, 2009, we re-addressed this question by identifying first primary diagnosed acute myeloid and lymphoid leukemia patients from the California Cancer Registry (CCR) and determined the cumulative incidences and risk factors for UE DVT, the rate of subsequent VTE and bleeding among those with incident UE DVT, and the impact of UE DVT on mortality.

## Methods

### Databases

The CCR is California's statewide population-based cancer surveillance system which maintains records about all malignancies diagnosed in California, with the exception of basal and squamous cell carcinoma of the skin. The CCR estimates that >98% of cancer diagnoses in California are captured. CCR data include the date of diagnosis, primary anatomical site, histologic type, American Joint Committee on Cancer stage, initial type of treatment, and basic demographic information.

The California Patient Discharge Database (PDD) and Emergency Department Utilization (EDU) contain records of all patients hospitalized in nonfederal hospitals or hospital-associated emergency departments in the state and are maintained by the Office of Statewide Health Planning and Development. Hospitals report up to 25 diagnoses and up to 21 procedures associated with each hospitalization, coded using the International Classification of Disease, Ninth Revision, Clinical Modification (ICD-9-CM) codes. A present-on-admission indicator is required for all PDD diagnoses. An encrypted version of the social security number is assigned to each patient which allows linkage of serial hospitalizations as well as linkages with other datasets.

### Patient Cohort

Using the CCR, we identified patients of all ages with a first primary diagnosis of acute leukemia from October 1, 2009 to December 31, 2014 and linked these patients with the PDD and EDU to identify incident UE DVT events using specific ICD-9-CM codes 453.82 to 453.87. All other ICD-9-CM codes for VTE, which comprise of pulmonary embolism (PE), LE DVT, and bleeding, are included in Supplementary Table S1 . Specific ICD-9-CM codes for UE DVT were updated at the beginning of the fourth quarter of 2009. Prior to this time, a physician would have to specifically mention the term phlebitis or thrombophlebitis to be abstracted as a specific UE DVT diagnosis and diagnoses such as DVT of an upper extremity or thoracic vein may have been coded as an LE DVT. 11 Therefore, we limited our analysis to after this time period. Patients with PE or LE DVT diagnosed prior to or at the time of leukemia diagnosis were excluded. In addition, chemotherapy was identified in CCR and/or PDD using ICD-9-CM code 99.25 in any procedure. Patients who were not treated with initial chemotherapy were excluded as we are unable to confirm the central venous catheter (CVC) status in this subgroup of patients. All patients who are treated with chemotherapy are assumed to have a CVC, an established risk factor for UE DVT. 12

**Table 1:** Baseline characteristics among treated California acute leukemia patients, 2009–2014

	AML		ALL	
**Variables**	***N*** ** = 3,252**	**%**	***N*** ** = 1,820**	**%**
*Leukemia subtype*				
AML-APL	406	12.5%	–	–
ALL-T cell	–	–	166	9.1%
ALL-B cell	–	–	1,650	90.7%
*Incident UE DVT*				
Yes	234	7.2%	114	6.3%
No	3,018	92.8%	1,706	93.7%
*Subsequent PE or LE DVT*				
PE Only	59	1.8%	40	2.2%
PE + DVT	20	0.6%	12	0.7%
DVT-proximal only	40	1.2%	27	1.5%
DVT-distal only	24	0.7%	12	0.7%
DVT-lower extremity, NOS only	2	0.1%	2	0.1%
*Gender*				
Male	1,805	55.5%	1,077	59.2%
Female	1,447	44.5%	743	40.8%
*Race/Ethnicity*				
Non-Hispanic White	1,755	54.0%	531	29.2%
African American	192	5.9%	87	4.8%
Hispanic	819	25.2%	1,018	55.9%
Asian/Pacific Islander	453	13.9%	169	9.3%
Other/Unknown	33	1.0%	15	0.8%
*Age at cancer diagnosis*				
<18	161	5.0%	770	42.3%
18–39	521	16.0%	449	24.7%
40–59	977	30.0%	372	20.5%
60–69	764	23.5%	146	8.0%
≥70	829	25.4%	83	4.6%
*HSCT*				
Yes	732	22.5%	335	18.4%
No	2,520	77.5%	1,485	81.6%
*Comorbidities (within 2 y prior)*				
Not available	897	27.6%	397	21.8%
0	204	6.3%	301	16.5%
1–2	869	26.7%	668	36.7%
≥3	1,282	39.4%	454	24.9%

Abbreviations: ALL, acute lymphoblastic leukemia; AML, acute myeloid leukemia; APL, acute promyelocytic leukemia; HSCT, hematopoietic stem cell transplantation; LE DVT, lower extremity deep vein thrombosis; NOS, not otherwise specified; PE, pulmonary embolism; UE DVT, upper extremity deep vein thrombosis.

### Covariates

From the CCR, we obtained patient demographics which included sex, race/ethnicity, age at diagnosis, neighborhood socioeconomic status, and health insurance at diagnosis or initial treatment. Children were defined as patients with a cancer diagnosis <18 years old, while adults were diagnosed at age ≥18 years. In addition, patients were categorized as acute promyelocytic leukemia (APL), a specific subtype of AML more prone to coagulopathy, and ALL patients were categorized as B-cell or T-cell. Comorbidities were captured up to 2 years prior to the acute leukemia diagnosis date. They were identified using codes that comprise the Elixhauser index, excluding cancer, 13 and categorized as no admissions in PDD within the 2 prior years of leukemia diagnosis (and thus no information), 0 comorbidities, 1 to 2 comorbidities, and ≥3 comorbidities. Hematopoietic stem cell transplant (HSCT) was identified in CCR and/or PDD using specific ICD-9-CM procedure codes ( Supplementary Table S2 ).

**Table 2:** Risk factors associated with upper extremity deep vein thrombosis development among treated California acute myeloid leukemia patients, 2009–2014

Variables	HR	95% CI	*p* -Value
*AML subtype*			
APL	1.36	(0.92, 2.02)	0.1271
No APL	Reference		
*Gender*			
Female	1.14	(0.86, 1.51)	0.3733
Male	Reference		
*Race/Ethnicity*			
Non-Hispanic White	Reference		
African American	1.64	(0.86, 3.11)	0.1316
Hispanic	0.99	(0.72, 1.35)	0.9273
Asian/Pacific Islander	0.78	(0.48, 1.28)	0.3319
*Age at cancer diagnosis*			
<50	Reference		
50–59	1.11	(0.77, 1.60)	0.5722
60–69	0.98	(0.69, 1.38)	0.8911
≥70	0.46	(0.25, 0.83)	0.0100
*HSCT* a			
Yes	1.95	(1.13, 3.36)	0.0158
No	Reference		

Abbreviations: AML, acute myeloid leukemia; APL, acute promyelocytic leukemia; CI, confidence interval; HR, hazard ratio; HSCT, hematopoietic stem cell transplantation.

Note: Multivariable cox proportional hazards model is stratified by comorbidities and adjusted for the competing risk of death using Fine and Gray methodology.

a HSCT is included as a time dependent covariate.

### Outcomes and Analysis Plan

Given the differences between treatment regimens and planned duration of therapy, analysis was stratified by the broad categories of acute leukemia, AML and ALL. Median follow-up time was calculated using the reverse Kaplan-Meier method. 14 15 We determined the 3- and 12-month cumulative incidence of first UE DVT, adjusted for the competing risk of death using the method of Fine and Gray. 16 The 3- and 12-month cumulative incidence of subsequent VTE (which included UE DVT, LE DVT, and PE) and bleeding after incident UE DVT was also calculated; time was calculated from date of UE DVT discharge to outcome event of interest, with death as a competing risk event or end of study. Because some patients with acute leukemia are frequently hospitalized, there is the possibility that a UE DVT code for an encounter soon after discharge from the incident UE DVT represented the same event, and not recurrence, as the treating physicians and abstractors would have considered it an active problem. To increase the specificity for UE DVT recurrence, a UE DVT code had to be in the principal position (the primary reason for inpatient admission). Or if acute leukemia was coded as the principal diagnosis, the acute UE DVT code had to be in the second position to be counted as a UE DVT recurrence. Using multivariable Cox proportional hazards regression models, adjusted for the competing risk of death, we identified risk factors for the development of incident UE DVT, the effect of incident UE DVT on the development of subsequent LE DVT and/or PE, the effect of incident UE DVT on subsequent bleeding, and the impact of incident UE DVT on leukemia-specific mortality and overall mortality. Analysis of the effect of incident UE DVT on subsequent bleeding was also adjusted for the competing risk of a subsequent VTE (LE DVT or PE) as this would likely modify the utilization of anticoagulation practices and therefore the risk of bleeding. Leukemia-specific mortality is measured from the date of diagnosis to the date of death from leukemia whereas overall survival considers death from all causes. Patients who died from causes other than leukemia were censored at the time of death in the analysis of leukemia-specific mortality. Patients alive at the study end date (December 31, 2014) were censored at this time or at the date of last known follow-up from the CCR. For all regression analyses, the proportional hazard assumption was assessed using Schoenfeld residuals. 17 Variables that violated the proportional hazard assumption were included as stratification variables. Subdistribution hazard ratios (HR) and adjusted HR are both presented as hazard ratios and 95% confidence intervals (CI) for simplicity. All analyses were done using SAS 9.4 and all statistical tests were two-sided; a *p* -value of less than 0.05 was considered statistically significant. This study was approved by the California Health and Human Services Agency Committee for the Protection of Human Subjects, and the University of California, Davis Institutional Review Boards.

## Results

We identified 5,072 patients between October 1, 2009 and December 31, 2014 with first primary diagnosis of acute leukemia who were treated with chemotherapy: 3,252 had AML and 1,820 had ALL. Baseline demographics are shown in Table 1 . Median follow-up times for AML and ALL were 30.9 months (95% CI: 29.4–32.2) and 32.0 months (95% CI: 29.9–33.9), respectively.

### Acute Myeloid Leukemia

Among 3,252 patients with AML, 406 (12.5%) had APL ( Table 1 ). Males comprised 55.5% and females 44.5% of patients. The majority of patients (54.0%) were non-Hispanic Whites, with Hispanics/Latino, Asians/Pacific Islanders, and African Americans contributing 25.2, 13.9, and 5.9%, respectively. Nearly half (48.9%) of the patients were 60 years or older and 66.1% of AML patients had one or more comorbidities. Approximately one-fifth (22.5%) underwent HSCT.

**Fig. 1:**
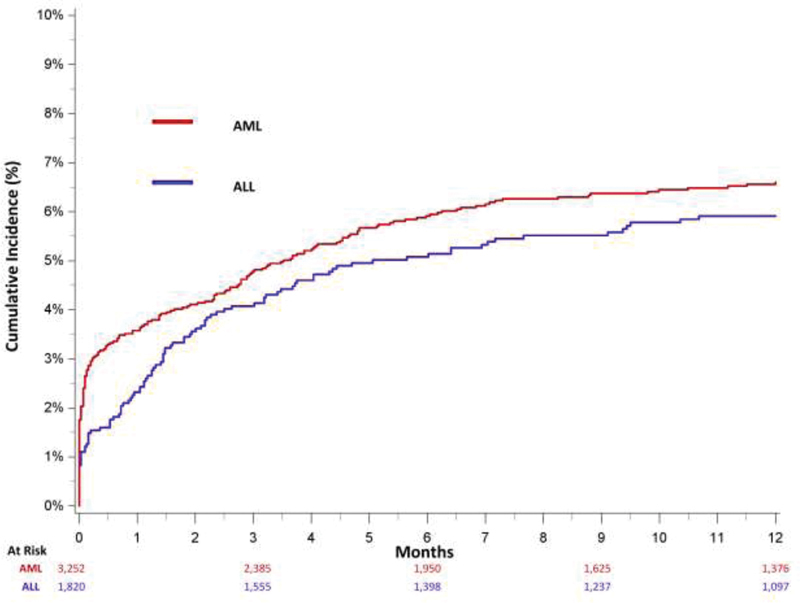
Cumulative incidence of upper extremity deep vein thrombosis, adjusted for the competing risk of death, among treated California acute leukemia patients, 2009 to 2014. AML, acute myeloid leukemia; ALL, acute lymphoblastic leukemia.

The 3- and 12-month cumulative incidences of UE DVT in AML were 4.8% (95% CI: 4.1–5.6) and 6.6% (95% CI: 5.8–7.5), respectively. Most (62.3%) incident UE DVTs occurred within the first 3 months of AML diagnosis ( Fig. 1 ). The multivariate analysis of risk factors for UE DVT in AML is shown in Table 2 . The risk of upper extremity DVT was not increased in APL compared with other subtypes of AML. Patients with age 70 and older were less likely to develop UE DVT (HR: 0.46; 95% CI: 0.25–0.83) than patients < 50 years. In addition, patients who underwent HSCT had approximately a twofold increased risk of UE DVT development compared with those who did not undergo HSCT (HR: 1.95; 95% CI: 1.13–3.36).

**Fig. 2:**
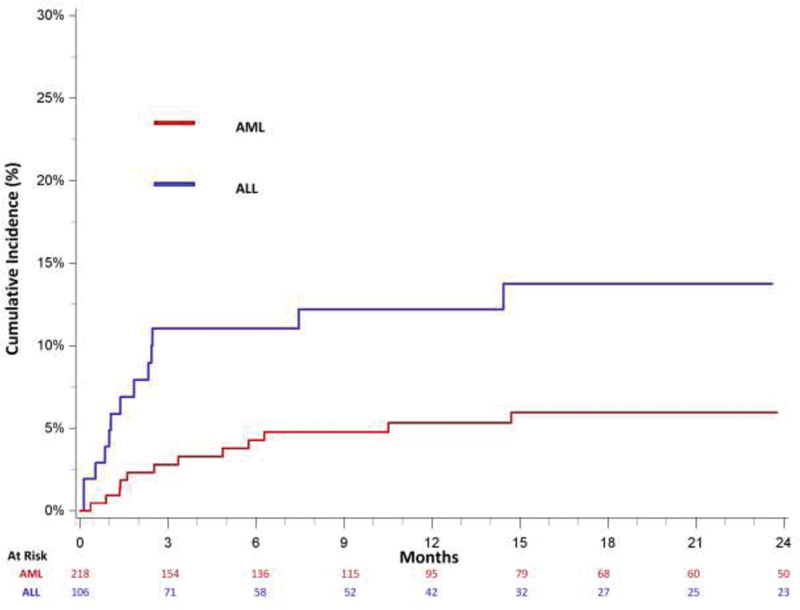
Cumulative incidence of subsequent venous thromboembolism, adjusted for the competing risk of death, among treated California acute leukemia patients with an upper extremity deep vein thrombosis, 2009 to 2014. AML, acute myeloid leukemia; ALL, acute lymphoblastic leukemia.

Fig. 2 shows the 3- and 12-month cumulative incidences of subsequent VTE after an incident UE DVT diagnosis, which were 2.8 and 5.3%, respectively. Approximately half (46.7%) of subsequent VTE events occurred within the first 3 months of incident UE DVT. The majority of subsequent VTE events were recurrent UE DVT with 3- and 12-month cumulative incidences of 2.3 and 3.4%, respectively. The 3- and 12-month cumulative incidences were 0.5 and 1.5% for subsequent LE DVT and 0 and 0.5% for subsequent PE, respectively. Upper extremity DVT was not a risk factor for development of subsequent PE or LE DVT in AML (HR: 0.68; 95% CI: 0.24–1.92) (data not shown).

**Fig. 3:**
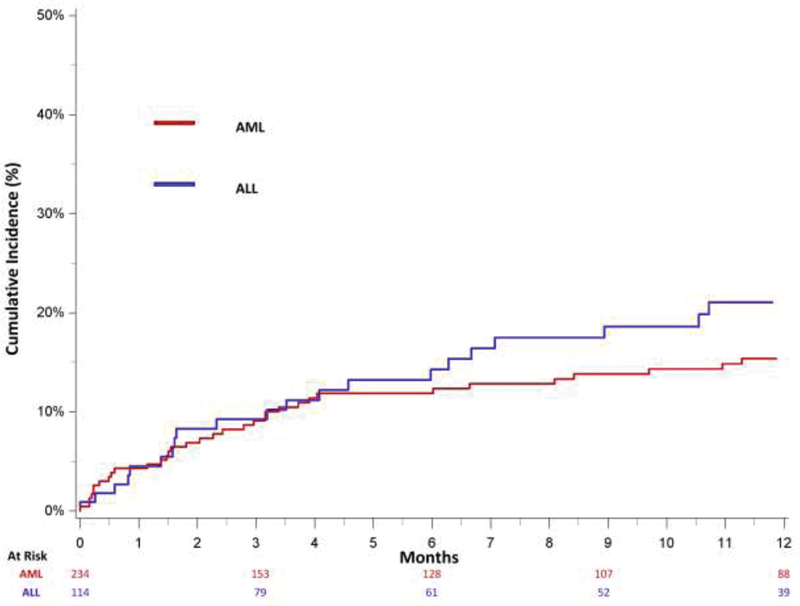
Cumulative incidence of subsequent bleeding, adjusted for the competing risk of subsequent VTE and death, among treated California acute leukemia patients with an upper extremity deep vein thrombosis, 2009 to 2014. AML, acute myeloid leukemia; ALL, acute lymphoblastic leukemia; VTE, venous thromboembolism.

The 3- and 12-month cumulative incidences of subsequent bleeding after an incident UE DVT diagnosis were 9.1 and 15.4%, respectively ( Fig. 3 ). Almost half (47.7%) of bleeding events occurred within the first 3 months of incident UE DVT. Among AML patients, the diagnosis of incident UE DVT was associated with an increased risk for subsequent bleeding (HR: 2.07; 95% CI: 1.60–2.68) ( Supplementary Table S3 ).

**Table 3:** Risk factors associated with leukemia-specific mortality among treated acute myeloid leukemia patients in California, 2009–2014

Variables	HR	95% CI	*p* -Value
*UE DVT* a			
Yes	1.42	(1.16, 1.73)	0.0006
No	Reference		
*PE ± LE DVT* a			
Yes	1.42	(1.13, 1.80)	0.0032
No	Reference		
*HSCT* a			
Yes	1.02	(0.60, 1.75)	0.9325
No	Reference		
*Gender*			
Female	0.86	(0.78, 0.95)	0.0045
Male	Reference		
*Race/Ethnicity*			
non-Hispanic White	Reference		
African American	0.96	(0.77, 1.20)	0.7154
Hispanic	0.97	(0.85, 1.11)	0.6898
Asian/Pacific Islander	1.15	(0.99, 1.33)	0.0685
*Age at diagnosis*			
<50	Reference		
50–59	1.47	(1.23, 1.75)	<0.0001
60–69	2.1	(1.77, 2.48)	<0.0001
≥70	3.64	(3.03, 4.37)	<0.0001
*Comorbidities (within 2 y prior)*			
Not available	1.19	(0.92, 1.53)	0.1775
0	Reference		
1–2	1.31	(1.02, 1.69)	0.0365
≥3	1.7	(1.33, 2.17)	<0.0001

Abbreviations: CI, confidence interval; HR, hazard ratio; HSCT, hematopoietic stem cell transplantation; LE DVT, lower extremity deep vein thrombosis; PE, pulmonary embolism; UE DVT, upper extremity deep vein thrombosis.

Note: Multivariable cox proportional hazards model is stratified by acute myeloid leukemia subtype.

a *UE DVT, PE* ± *LE DVT, and SCT are included as time-dependent covariates.*

A diagnosis of incident UE DVT was associated with increased leukemia-specific mortality in AML (HR: 1.42; 95% CI: 1.16–1.73) ( Table 3 ). A diagnosis of PE and/or LE DVT, increasing age at AML diagnosis, and increasing comorbidities was also associated with increased leukemia-specific mortality. Female gender was associated with decreased mortality (HR: 0.86; 95% CI: 0.78–0.95). Upper extremity DVT diagnosis was associated with increased overall mortality in AML (HR: 1.35; 95% CI: 1.11–1.63) (data not shown).

### Acute Lymphoblastic Leukemia

Among the 1,820 patients with ALL, 1,650 (90.7%) had B-cell ALL and 166 (9.1%) had T-cell ALL ( Table 1 ). Fifty-nine percent of patients were male and 41% were female. The majority of patients with ALL (55.9%) were Hispanics, with non-Hispanic Whites, Asians/Pacific-Islanders, and African Americans contributing 29.2, 9.3, and 4.8%, respectively, the demographics of which are similar to prior epidemiologic studies in ALL. 18 Approximately half (58.6%) of patients were less than 29 years, consistent with ALL being primarily a disease of the pediatric and adolescent and young adult population. 19 One or more co-morbidities were present in 61.6% of the patients and 18.4% underwent HSCT.

The 3- and 12-month cumulative incidences of UE DVT in ALL were 4.1% (95% CI: 3.2–5.1) and 5.9% (95% CI: 4.9–7.1), respectively. Most (64.1%) of UE DVT occurred within the first 3 months of diagnosis ( Fig. 1 ). The multivariate analysis of risk factors associated with UE DVT is shown in Table 4 . Upper extremity DVT risk was similar among both subtypes of ALL. Older age at diagnosis was associated with increased incident UE DVT in all age groups compared with <18 years, except those aged 30 to 39 years where the association was borderline. HSCT was not associated with increased risk of incident UE DVT in ALL although the cohort size was small.

**Table 4:** Risk factors associated with upper extremity deep vein thrombosis development among treated California acute lymphoblastic leukemia patients, 2009–2014

Variables	HR	95% CI	*p* -Value
*Gender*			
Female	1.01	(0.68, 1.49)	0.9734
Male	Reference		
*Race/Ethnicity*			
Non-Hispanic White	Reference		
African American	0.36	(0.12, 1.02)	0.0553
Hispanic	0.68	(0.45, 1.03)	0.0685
Asian/Pacific Islander	0.83	(0.39, 1.75)	0.6231
Age at diagnosis			
<18	Reference		
18–29	4.72	(2.54, 8.75)	<0.0001
30–39	2.42	(0.97, 6.02)	0.0587
40–49	3.39	(1.65, 6.96)	0.0009
50–59	3.11	(1.59, 6.06)	0.0009
≥60	3.37	(1.68, 6.78)	0.0007
*HSCT* a			
Yes	1.55	(0.66, 3.65)	0.3182
No	Reference		

Abbreviations: CI, confidence interval; HR, hazard ratio; HSCT, hematopoietic stem cell transplantation.

Note: Multivariable cox proportional hazards model is stratified by acute lymphoblastic leukemia subtype and comorbidities and adjusted for the competing risk of death using Fine and Gray methodology.

a HSCT is included as a time dependent covariate.

The 3- and 12-month cumulative incidences of subsequent VTE after an incident UE DVT diagnosis were 11.0 and 12.2% for ALL, respectively ( Fig. 2 ). Eighty percent of subsequent VTEs occurred within the first 3 months of incident UE DVT diagnosis and most were UE DVT which had 3- and 12-month cumulative incidences of 6.0 and 7.2%., respectively. The 3-month cumulative incidences were 1.0% for subsequent LE DVT and 4.0% for subsequent PE, respectively. Twelve-month cumulative incidences for subsequent LE DVT and PE were unchanged compared with the incidence at 3 months. Among ALL patients, UE DVT was not associated with an increased risk for subsequent incident PE or LE DVT development (HR: 1.84; 95% CI: 0.76–4.42) (data not shown).

Fig. 3 shows the 3- and 12-month cumulative incidences of subsequent bleeding after an incident UE DVT diagnosis, which was 9.2 and 21.1%, respectively Thirty-six percent of bleeding events occurred during the first 3 months after UE DVT diagnosis. Among ALL patients, a diagnosis of UE DVT was associated with an increased risk for subsequent bleeding (HR: 1.62; 95% CI: 1.02–2.57) ( Supplementary Table S4 ).

A diagnosis of incident UE DVT was associated with increased leukemia-specific mortality in ALL (HR: 1.80; 95% CI: 1.31–2.47) ( Table 5 ). A diagnosis of PE and/or LE DVT and increasing age of diagnosis were also associated with increased leukemia-specific mortality. Upper extremity DVT diagnosis was associated with increased overall mortality in ALL (HR: 1.74; 95% CI: 1.28–2.36) (data not shown).

**Table 5:** Risk factors associated with leukemia-specific mortality among treated acute lymphoblastic leukemia patients in California, 2009–2014

Variables	HR	95% CI	*p* -Value
*UE DVT* a			
Yes	1.80	(1.31, 2.47)	0.0003
No	Reference		
*PE ± LE DVT* a			
Yes	1.41	(1.01, 1.98)	0.0427
No	Reference		
*HSCT* a			
Yes	1.21	(0.44, 3.33)	0.7059
No	Reference		
*Gender*			
Female	0.83	(0.69, 1.01)	0.0624
Male	Reference		
*Race/Ethnicity*			
Non-Hispanic (NH) White	Reference		
African American (vs. NH White)	1.11	(0.70, 1.75)	0.6647
Hispanic	1.02	(0.81, 1.29)	0.8589
Asian/PI	1.17	(0.84, 1.64)	0.361
*Age at diagnosis*			
<18	Reference		
18–29	3.21	(2.25, 4.60)	<0.0001
30–39	5.37	(3.57, 8.09)	<0.0001
40–49	6.51	(4.36, 9.73)	<0.0001
50–59	6.03	(3.99, 9.12)	<0.0001
≥60	6.71	(4.31, 10.42)	<0.0001
*Comorbidities (within 2 y prior)*			
Not available	1.47	(0.95, 2.27)	0.0855
0	Reference		
1–2 comorbidities	2.13	(1.43, 3.18)	0.0002
≥3	2.99	(1.99, 4.49)	<0.0001

Abbreviations: CI, confidence interval; HR, hazard ratio; HSCT, hematopoietic stem cell transplantation; LE DVT, lower extremity deep vein thrombosis; PE, pulmonary embolism; PI, Pacific Islanders; UE DVT, upper extremity deep vein thrombosis.

Note: Multivariable cox proportional hazards model is stratified by initial course of treatment-radiation.

a *UE DVT, PE* ± *LE DVT and transplant are included as time-dependent covariates.*

## Discussion

In this large population-based study, we observed that the 12-month cumulative incidence of UE DVT was appreciable at 6.6% for AML and 5.9% for ALL, with most UE DVT events occurring within the first 3 months of acute leukemia diagnosis. HSCT was a risk factor for UE DVT development in AML patients while adult ALL patients were at higher risk for UE DVT development compared with children. Twelve-month cumulative incidences of subsequent VTE after an incident UE DVT diagnosis were 5.3% for AML and 12.2% for ALL; the majority of subsequent VTE events were recurrent UE DVTs. While UE DVT was not a risk factor for development of subsequent PE or LE DVT in AML or ALL, it was associated with an increased risk for subsequent bleeding in both AML and ALL, suggesting these patients may have received anticoagulation. A diagnosis of incident UE DVT was associated with increased leukemia-specific and overall mortality in both acute leukemia subtypes.

The 12-month cumulative incidence of UE DVT was significantly higher in our study than previously published reports. 6 20 Our prior analysis in California from 1993 to 1999 reported 12-month cumulative incidences of UE DVT at 1.5% for AML and 0.9% for ALL, but was limited by lack of specific diagnostic codes for UE DVT (at the time a physician would have to specifically mention the term phlebitis or thrombophlebitis to be abstracted as a specific UE DVT diagnosis). 6 A single institution retrospective study of acute leukemia patients treated with chemotherapy between 1999 and 2005 reported UE DVT incidences of 2.5% with AML and 2.5% with ALL after an overall median follow-up of 13.9 months. 20 While changes in coding that more accurately captured a diagnosis of UE DVT after 2009 may contribute to the higher incidence we observed, 11 the incidence may also be higher in our study because of more accurate diagnostic methods over the past decade 21 or because we limited our analysis to patients treated with chemotherapy, a group at higher risk for VTE. 22 23

Among AML patients, HSCT was a risk factor for UE DVT development. This finding is consistent with a meta-analysis of six studies evaluating the association between leukemia and VTE which observed that HSCT recipients had the highest incidences of VTE among all acute leukemia patients. 24 In the current study, UE DVT incidence was not increased in APL compared with other subtypes of AML, an unexpected finding as APL is known to have an increased inherent risk of thrombosis and coagulopathy compared with other subtypes of leukemia. 25 26

In ALL, adults were found to have a higher risk for UE DVT development compared with children, which is consistent with prior data showing that VTE incidence in the general population increases with age. 27 28 In contrast to AML, HSCT was not associated with increased risk of incident UE DVT in ALL; however, the cohort size was small. Other established risk factors for UE DVT development in ALL, which were unavailable data in the current study, include the presence of mediastinal masses which has been reported in 10 to 15% of children at the time of ALL diagnosis, and exposure to a combination of steroids and asparaginase during treatment. 29 30 31 32

In our study, African Americans and Asian/Pacific Islanders had a similar risk of incident UE DVT compared with non-Hispanic Whites for both AML and ALL. This is in contrast to the higher risk of VTE in African Americans and lower risk in Asian/Pacific Islanders commonly seen with other malignancies. 4 33 Other known risk factors for VTE development include the underlying coagulopathy and prothrombotic environment characteristic of acute leukemia. 34 35 In addition, the presence of CVCs which is nearly ubiquitous to all acute leukemia patients who receive treatment also increase VTE risk. 20 36 A prospective cohort study of patients with solid tumors and implanted ports observed an appreciable CVC-associated VTE incidence of 3.8% and found 30% of all VTE events to be catheter associated. 37

Our analysis found the 12-month cumulative incidences of VTE after incident UE DVT to be considerable at 5.3% for AML and 12.2% for ALL. The most common subsequent VTE subtype was recurrent UE DVT. Nevertheless, the cumulative incidences of subsequent lower extremity DVT and PE were still appreciable after incident UE DVT. These findings are supported by a single institution retrospective review which noted a 12-month incidence of recurrent VTE in acute leukemia patients to be as high as 16.6%. 38 Another single institution review found 30% of acute leukemia patients with incident VTE to have recurrent VTE. 39 Our analysis, in addition to previously mentioned studies, supports that VTE in itself is an important risk factor for subsequent VTE development in acute leukemia.

The 12-month cumulative incidences of bleeding after incident UE DVT were considerable at 15.4% for AML and 21.2% for ALL; these incidences are likely underestimates of the overall bleeding risk in acute leukemia patients, as we excluded bleeding events which occurred during the same hospitalization as incident UE DVT diagnosis. Furthermore, isolated UE DVT was associated with an increased risk of subsequent bleeding in both acute leukemia subtypes. Although our databases do not include medication data, we suspect the increased risk of bleeding is likely due to use of anticoagulation. These findings are supported by a single institution retrospective review which noted substantially higher bleeding rates among patients with hematologic malignancies and VTE receiving anticoagulation compared with those without anticoagulation at 27 versus 3%, respectively. 40 In addition, a systemic review of 13 observational studies investigating VTE treatment patterns among 5,359 acute leukemia patients revealed a high rate of anticoagulation use at 73% among those diagnosed with VTE. 41 Other single institution studies similarly report high rates of anticoagulation use of up to 90% among leukemia patients diagnosed with VTE and variable bleeding rates (5–77%) among anticoagulated patients. 42 43 44

A diagnosis of incident UE DVT was an independent predictor for increased leukemia-specific mortality and overall mortality in both acute leukemia subtypes. However, UE DVT was not an independent risk factor for subsequent PE or LE DVT for either leukemia subtype. These mortality model findings are consistent with VTE being associated with increased mortality in cancer patients. 45 46 However, most prior studies examining the effect of VTE on mortality in cancer patients are limited to PE and proximal LE DVT in their analysis, 47 48 while expert opinion has historically attributed UE DVT in malignancy to be less precarious. 49 50 The increased risk of mortality associated with isolated UE DVT in our study is consistent with a recent study showing that cancer patients with isolated distal LE DVT have similar rates of VTE recurrence and mortality as compared with those with proximal DVT. 10 This suggests that in cancer patients, VTE regardless of location has a significant impact on mortality. A possible explanation for the effect of UE DVT on mortality is that it may be a surrogate for disease severity rather than the ultimate cause of demise in patients with leukemia. This hypothesis is supported by a systemic review of UE DVT in patients with and without malignancy that found the high mortality in UE DVT patients is often due to their underlying diseases rather than the UE DVT or its complications. 51 The high cumulative incidence of bleeding after an isolated UE DVT and the increased risk of bleeding associated with UE DVT may have also contributed to the increase in mortality, particularly in elderly patients.

There are limitations to our study. Our analysis was limited to those undergoing treatment with systemic chemotherapy as it was assumed that all leukemia patients undergoing treatment would have a CVC. It is not clear if the findings would be generalizable to those patients with acute leukemia not receiving intravenous chemotherapy, especially since 2017 when the use of oral drugs targeting various epigenetic mutations was approved for acute leukemia treatment. 52 Although our registries were limited to the inpatient and emergency department settings, most VTE events were likely captured as our study ended prior to the introduction of outpatient chemotherapy for AML and given the high acuity of leukemia patients, most diagnosed with VTE would likely have been admitted or evaluated in the emergency department. Another limitation to our analysis is the lack of data on the management of UE DVT in terms of anticoagulation use (prophylactic and therapeutic), timing of CVC removal, or inferior vena cava filter placement, all factors which can affect recurrent VTE development and bleeding.

Strengths of this study include the large population-based cohort of 5,072 patients with acute leukemia which allowed for a robust multivariate analysis of potential risk factors and outcomes; previous studies were primarily limited to single institution studies or literature reviews. In addition, this is one of the few studies that specifically investigates isolated UE DVT, as previous studies generally either excluded UE DVT from their VTE analysis or combined the incidence with PE or LE DVT. Lastly, ICD-9-CM codes were updated in October 2009 at the start of our cohort which likely provides a more accurate assessment of the cumulative incidence of UE DVT as compared with previous studies using administrative and/or primary data relying on ICD-9-CM coding.

## Conclusion

In one of the largest studies to date examining the cumulative incidence of UE DVT in acute leukemia, we observed the 3- and 12-month cumulative incidences of UE DVT in both AML and ALL to be higher than previously reported. As previously shown, added to the incidence of LE DVT and PE in these patients, the incidence of VTE in patients with acute leukemia is similar to that seen with many solid tumors. 6 53 In addition, isolated UE DVT, absent of other concomitant VTE, was associated with increased mortality, raising the possibility that UE DVT in itself may be a marker of disease severity. This study helps identify the cumulative incidence and risk factors associated with UE DVT and subsequent VTE and bleeding events in patients with acute leukemia. Prospective studies investigating the use and complications of prophylactic and therapeutic anticoagulation are needed in this high-risk population.

## References

[1] Noble S Pasi J Epidemiology and pathophysiology of cancer-associated thrombosis Br J Cancer 2010 102 01 S2 S9 20386546 10.1038/sj.bjc.6605599 PMC3315367

[2] Timp J F Braekkan S K Versteeg H H Cannegieter S C Epidemiology of cancer-associated venous thrombosis Blood 2013 122 10 1712 1723 23908465 10.1182/blood-2013-04-460121

[3] Khorana A A Francis C W Culakova E Kuderer N M Lyman G H Frequency, risk factors, and trends for venous thromboembolism among hospitalized cancer patients Cancer 2007 110 10 2339 2346 17918266 10.1002/cncr.23062

[4] Chew H K Wun T Harvey D Zhou H White R H Incidence of venous thromboembolism and its effect on survival among patients with common cancers Arch Intern Med 2006 166 04 458 464 16505267 10.1001/archinte.166.4.458

[5] Levitan N Dowlati A Remick S C Rates of initial and recurrent thromboembolic disease among patients with malignancy versus those without malignancy. Risk analysis using Medicare claims data Medicine (Baltimore) 1999 78 05 285 291 10499070 10.1097/00005792-199909000-00001

[6] Ku G H White R H Chew H K Harvey D J Zhou H Wun T Venous thromboembolism in patients with acute leukemia: incidence, risk factors, and effect on survival Blood 2009 113 17 3911 3917 19088376 10.1182/blood-2008-08-175745 PMC2673120

[7] Blom J W Doggen C J Osanto S Rosendaal F R Malignancies, prothrombotic mutations, and the risk of venous thrombosis JAMA 2005 293 06 715 722 15701913 10.1001/jama.293.6.715

[8] RIETE Investigators Muñoz F J Mismetti P Poggio R Clinical outcome of patients with upper-extremity deep vein thrombosis: results from the RIETE Registry Chest 2008 133 01 143 148 17925416 10.1378/chest.07-1432

[9] Valerio L Ambaglio C Barone M Recurrence risk after first symptomatic distal versus proximal deep vein thrombosis according to baseline risk factors TH Open 2019 3 01 e58 e63 31249983 10.1055/s-0039-1683374 PMC6524909

[10] Poudel S K Park D Y Jia X Clinical outcomes of isolated distal deep vein thrombosis versus proximal venous thromboembolism in cancer patients: the Cleveland Clinic experience J Thromb Haemost 2020 18 03 651 659 31808607 10.1111/jth.14700

[11] Fang M C Fan D Sung S H Validity of using inpatient and outpatient administrative codes to identify acute venous thromboembolism: the CVRN VTE study Med Care 2017 55 12 e137 e143 29135777 10.1097/MLR.0000000000000524 PMC5125903

[12] Lee J A Zierler B K Zierler R E The risk factors and clinical outcomes of upper extremity deep vein thrombosis Vasc Endovascular Surg 2012 46 02 139 144 22328450 10.1177/1538574411432145

[13] Schoenman J A Sutton J P Elixhauser A Love D Understanding and enhancing the value of hospital discharge data Med Care Res Rev 2007 64 04 449 468 17684112 10.1177/1077558707301963

[14] Clark T G Bradburn M J Love S B Altman D G Survival analysis part I: basic concepts and first analyses Br J Cancer 2003 89 02 232 238 12865907 10.1038/sj.bjc.6601118 PMC2394262

[15] Schemper M Smith T L A note on quantifying follow-up in studies of failure time Control Clin Trials 1996 17 04 343 346 8889347 10.1016/0197-2456(96)00075-x

[16] Fine J P Gray R J A proportional hazards model for the subdistribution of a competing risk J Am Stat Assoc 1999 94 446 496 509

[17] Schoenfeld D Chi-squared goodness-of-fit tests for the proportional hazards regression model Biometrika 1980 67 01 145 153

[18] Zhao Y Wang Y Ma S Racial differences in four leukemia subtypes: comprehensive descriptive epidemiology Sci Rep 2018 8 01 548 29323237 10.1038/s41598-017-19081-4 PMC5765036

[19] Ward E DeSantis C Robbins A Kohler B Jemal A Childhood and adolescent cancer statistics, 2014 CA Cancer J Clin 2014 64 02 83 103 24488779 10.3322/caac.21219

[20] Vu K Luong N V Hubbard J A retrospective study of venous thromboembolism in acute leukemia patients treated at the University of Texas MD Anderson Cancer Center Cancer Med 2015 4 01 27 35 25487644 10.1002/cam4.332 PMC4312115

[21] Di Nisio M Van Sluis G L Bossuyt P M Büller H R Porreca E Rutjes A W Accuracy of diagnostic tests for clinically suspected upper extremity deep vein thrombosis: a systematic review J Thromb Haemost 2010 8 04 684 692 20141579 10.1111/j.1538-7836.2010.03771.x

[22] Lyman G H Eckert L Wang Y Wang H Cohen A Venous thromboembolism risk in patients with cancer receiving chemotherapy: a real-world analysis Oncologist 2013 18 12 1321 1329 24212499 10.1634/theoncologist.2013-0226 PMC3868427

[23] Ferroni P Riondino S Guadagni F Roselli M Impact of chemotherapy on venous thromboembolism: comment to: regional lymph node metastases are a strong risk factor for venous thromboembolism: results from the Vienna Cancer and Thrombosis Study Haematologica 2013 98 12 e153 e154 24323987 10.3324/haematol.2013.092528 PMC3856977

[24] Wu Y Y Tang L Wang M H Leukemia and risk of venous thromboembolism: a meta-analysis and systematic review of 144 studies comprising 162,126 patients Sci Rep 2017 7 01 1167 28446766 10.1038/s41598-017-01307-0 PMC5430898

[25] Ziegler S Sperr W R Knöbl P Symptomatic venous thromboembolism in acute leukemia. Incidence, risk factors, and impact on prognosis Thromb Res 2005 115(1-2):59 64 15567454 10.1016/j.thromres.2004.07.016

[26] De Stefano V Sorà F Rossi E The risk of thrombosis in patients with acute leukemia: occurrence of thrombosis at diagnosis and during treatment J Thromb Haemost 2005 3 09 1985 1992 16102104 10.1111/j.1538-7836.2005.01467.x

[27] Engbers M J van Hylckama Vlieg A Rosendaal F R Venous thrombosis in the elderly: incidence, risk factors and risk groups J Thromb Haemost 2010 8 10 2105 2112 20629943 10.1111/j.1538-7836.2010.03986.x

[28] Stein P D Kayali F Olson R E Incidence of venous thromboembolism in infants and children: data from the National Hospital Discharge Survey J Pediatr 2004 145 04 563 565 15480387 10.1016/j.jpeds.2004.06.021

[29] Ravindranath Y Kaplan J Zuelzer W W Significance of mediastinal mass in acute lymphoblastic leukemia Pediatrics 1975 55 06 889 893 1094403

[30] Goyal G Bhatt V R L-asparaginase and venous thromboembolism in acute lymphocytic leukemia Future Oncol 2015 11 17 2459 2470 26274336 10.2217/fon.15.114 PMC4976870

[31] Truelove E Fielding A K Hunt B J The coagulopathy and thrombotic risk associated with L-asparaginase treatment in adults with acute lymphoblastic leukaemia Leukemia 2013 27 03 553 559 23099335 10.1038/leu.2012.290

[32] Levy-Mendelovich S Barg A A Kenet G Thrombosis in pediatric patients with leukemia Thromb Res 2018 164 01 S94 S97 29703491 10.1016/j.thromres.2018.01.019

[33] White R H Keenan C R Effects of race and ethnicity on the incidence of venous thromboembolism Thromb Res 2009 123 04 S11 S17 19303496 10.1016/S0049-3848(09)70136-7

[34] Payne J H Vora A J Thrombosis and acute lymphoblastic leukaemia Br J Haematol 2007 138 04 430 445 17608766 10.1111/j.1365-2141.2007.06677.x

[35] Crespo-Solís E Thrombosis and acute leukemia Hematology 2012 17 01 S169 S173 22507812 10.1179/102453312X13336169156852

[36] Wun T White R H Venous thromboembolism in patients with acute leukemia, lymphoma, and multiple myeloma Thromb Res 2010 125 02 S96 S102 20434017 10.1016/S0049-3848(10)70024-4

[37] ONCOCIP Investigators Decousus H Bourmaud A Fournel P Cancer-associated thrombosis in patients with implanted ports: a prospective multicenter French cohort study (ONCOCIP) Blood 2018 132 07 707 716 29980524 10.1182/blood-2018-03-837153

[38] Luong N V Kroll M H Vu K Recurrence of venous thromboembolism among adults acute leukemia patients treated at the University of Texas MD Anderson Cancer Center: incidence and risk factors Thromb Res 2017 156 14 19 28577388 10.1016/j.thromres.2017.05.019

[39] Wang Y Cantor N Carrier M Characteristics of venous thromboembolism in patients with acute leukemia Blood 2017 130 01 3869 3869

[40] Houghton D E Key N S Zakai N A Laux J P Shea T C Moll S Analysis of anticoagulation strategies for venous thromboembolism during severe thrombocytopenia in patients with hematologic malignancies: a retrospective cohort Leuk Lymphoma 2017 58 11 2573 2581 28393576 10.1080/10428194.2017.1306644 PMC8211440

[41] Ahrari A Al-Ani F Wang Y P Lazo-Langner A Treatment of venous thromboembolism in acute leukemia: a systematic review Thromb Res 2019 178 1 6 30921533 10.1016/j.thromres.2019.03.014

[42] Bleker S M van Es N Kleinjan A Current management strategies and long-term clinical outcomes of upper extremity venous thrombosis J Thromb Haemost 2016 14 05 973 981 26866515 10.1111/jth.13291

[43] Scott M Hibino M Cavalier M Incidence of bleeding events in acute myeloid leukemia (AML) patients receiving anticoagulation therapy for treatment of deep vein thrombosis (DVT) or pulmonary embolism (PE) who are also undergoing induction or re-induction therapy Blood 2017 130 01 2091

[44] Kelkar A H Dhanarajan A R Kelkar M A Wingard J R Bleeding and thrombosis outcomes in patients with acute leukemia J Clin Oncol 2020 38 15 e19501

[45] Khorana A A Venous thromboembolism and prognosis in cancer Thromb Res 2010 125 06 490 493 20097409 10.1016/j.thromres.2009.12.023 PMC2878879

[46] Sørensen H T Mellemkjaer L Olsen J H Baron J A Prognosis of cancers associated with venous thromboembolism N Engl J Med 2000 343 25 1846 1850 11117976 10.1056/NEJM200012213432504

[47] Tagalakis V Patenaude V Kahn S R Suissa S Incidence of and mortality from venous thromboembolism in a real-world population: the Q-VTE study cohort Am J Med 2013 126 09 8.32E15 8.32E23 10.1016/j.amjmed.2013.02.024 23830539

[48] Søgaard K K Schmidt M Pedersen L Horváth-Puhó E Sørensen H T 30-year mortality after venous thromboembolism: a population-based cohort study Circulation 2014 130 10 829 836 24970783 10.1161/CIRCULATIONAHA.114.009107

[49] Tilney M L Griffiths H J Edwards E A Natural history of major venous thrombosis of the upper extremity Arch Surg 1970 101 06 792 796 5489306 10.1001/archsurg.1970.01340300148026

[50] Gloviczki P Kazmier F J Hollier L H Axillary-subclavian venous occlusion: the morbidity of a nonlethal disease J Vasc Surg 1986 4 04 333 337 3761474 10.1067/mva.1986.avs0040333

[51] Heil J Miesbach W Vogl T Bechstein W O Reinisch A Deep vein thrombosis of the upper extremity Dtsch Arztebl Int 2017 114 14 244 249 28446351 10.3238/arztebl.2017.0244 PMC5415909

[52] Winer E S Stone R M Novel therapy in acute myeloid leukemia (AML): moving toward targeted approaches Ther Adv Hematol 2019 10 1 8 10.1177/2040620719860645 PMC6624910 31321011

[53] Yıldız A Albayrak M Pala Ç The incidence and risk factors of thrombosis and the need for thromboprophylaxis in lymphoma and leukemia patients: a 9-year single-center experience J Oncol Pharm Pract 2020 26 02 386 396 31156054 10.1177/1078155219851540

