# Supplementary material for: Incidence of Upper Extremity Deep Vein Thrombosis in Acute Leukemia and Effect on Mortality
Source: TH Open. 2020 Oct 28;4(4):e309–17. doi: 10.1055/s-0040-1718883 (PMC7593117; doi:10.1055/s-0040-1718883)
Supplement: Supplementary file 1 — Supplementary Material [file 10-1055-s-0040-1718883-s200046.pdf]

Supplementary Table S1 International Classification of Disease, Ninth Revision, Clinical Modification (ICD-9-CM) codes used to identify venous thromboembolism (VTE) and bleeding

Code	Category	Description
VTE		
415.11[a]	PE	Iatrogenic pulmonary embolism and infarction
415.13[a]	PE	Saddle embolus of pulmonary artery
415.19[a]	PE	Other pulmonary embolism and infarction
673.2[a]	PE	Obstetrical blood clot embolism
451.11	LE DVT	Thrombophlebitis of deep vessels of lower extremities, femoral vein
451.19	LE DVT	Thrombophlebitis of deep vessel of lower extremities, other
451.81	LE DVT	Thrombophlebitis of other sites, iliac vein
453.2	LE DVT	Venous thrombosis of inferior vena cava
453.4	LE DVT	Acute venous thrombosis of unspecified deep vessels of lower extremity
453.41[a]	LE DVT	Acute venous thrombosis of deep vessels of proximal lower extremity
453.42[a]	LE DVT	Acute venous thrombosis of deep vessels of distal lower extremity
453.8	LE DVT	Acute venous thrombosis of other specified veins (eliminated October 1, 2009)
453.89	LE DVT	Acute venous thrombosis of other specified veins
453.9	LE DVT	Acute venous thrombosis of unspecified site
671.31[a]	LE DVT	DVT antepartum—delivered
671.33[a]	LE DVT	DVT antepartum
671.42[a]	LE DVT	DVT postpartum—delivered
671.44[a]	LE DVT	DVT postpartum
Bleeding		
430	ICH	Nontraumatic subarachnoid hemorrhage
431	ICH	Nontraumatic intracerebral hemorrhage
432	ICH	Nontraumatic extradural hemorrhage
432.1	ICH	Subdural hemorrhage
432.9	ICH	Intracranial hemorrhage, NOS
456	GI	Esophagus varices w/bleed
456.2	GI	Bleed esophagus varices other disease
530.7	GI	Mallory-Weiss syndrome

(*Continued*)

Code	Category	Description
Bleeding		
530.82	GI	Esophageal hemorrhage (Begin 1993)
531	GI	Acute gastric ulcer w/hemorrhage, w/out obstruction
531.01	GI	Acute gastric ulcer w/hemorrhage, w/obstruction
531.2	GI	Acute gastric ulcer w/hemorrhage, w/perforation
53121	GI	Acute gastric ulcer w/hemorrhage, w/perforation, and obstruction
531.4	GI	Chronic gastric ulcer w/hemorrhage, w/out obstruction
531.41	GI	Chronic gastric ulcer w/hemorrhage, w/obstruction
531.6	GI	Chronic gastric ulcer w/hemorrhage, w/perforation
531.61	GI	Chronic gastric ulcer w/hemorrhage, w/perforation, and obstruction
532	GI	Acute duodenal ulcer w/hemorrhage, w/out obstruction
532.01	GI	Acute duodenal ulcer w/hemorrhage, w/obstruction
532.2	GI	Acute duodenal ulcer w/hemorrhage, w/perforation
532.21	GI	Acute duodenal ulcer w/hemorrhage, w/perforation, and obstruction
532.4	GI	Chronic duodenal ulcer w/hemorrhage, w/out obstruction
532.41	GI	Chronic duodenal ulcer w/hemorrhage, w/obstruction
532.6	GI	Chronic duodenal ulcer w/hemorrhage, w/perforation
532.61	GI	Chronic duodenal ulcer w/hemorrhage, w/perforation, and obstruction
533	GI	Acute peptic ulcer w/hemorrhage, w/out obstruction
533.01	GI	Acute peptic ulcer w/hemorrhage, w/obstruction
533.2	GI	Acute peptic ulcer w/hemorrhage, w/perforation
533.21	GI	Acute peptic ulcer w/hemorrhage, w/perforation, and obstruction
53340	GI	Chronic peptic ulcer w/hemorrhage, w/out obstruction

(*Continued*)

Code	Category	Description
**Bleeding**		
533.41	GI	Chronic peptic ulcer w/hemorrhage, w/obstruction
533.6	GI	Chronic peptic ulcer w/hemorrhage, w/perforation
533.61	GI	Chronic peptic ulcer w/hemorrhage, w/perforation, and obstruction
534	GI	Acute gastrojejunal ulcer w/hemorrhage, w/out obstruction
534.01	GI	Acute gastrojejunal ulcer w/hemorrhage, w/obstruction
534.2	GI	Acute gastrojejunal ulcer w/hemorrhage, w/perforation
534.21	GI	Acute gastrojejunal ulcer w/hemorrhage, w/perforation, and obstruction
534.4	GI	Chronic gastrojejunal ulcer w/hemorrhage, w/out obstruction
534.41	GI	Chronic gastrojejunal ulcer w/hemorrhage, w/obstruction
534.6	GI	Chronic gastrojejunal ulcer w/hemorrhage, w/perforation
534.61	GI	Chronic gastrojejunal ulcer w/hemorrhage, w/perforation, and obstruction
535.01	GI	Acute gastritis w/hemorrhage
535.11	GI	Atrophic gastritis w/hemorrhage
535.21	GI	Gastric mucosal hypertrophy w/hemorrhage
535.31	GI	Alcoholic gastritis w/hemorrhage
535.41	GI	Other specified gastritis w/hemorrhage
535.51	GI	Unspecified gastritis and gastroduodenitis w/hemorrhage
535.61	GI	Duodenitis w/hemorrhage
537.83	GI	Angiodysplasia of stomach and duodenum w/hemorrhage
562.02	GI	Diverticulosis of small intestine w/hemorrhage
562.03	GI	Diverticulitis of small intestine w/hemorrhage
562.12	GI	Diverticulosis of colon w/hemorrhage
562.13	GI	Diverticulitis of colon w/hemorrhage
569.3	GI	Rectal and anal hemorrhage
569.85	GI	Angiodysplasia of intestine w/hemorrhage
578	GI	Hematemesis

(*Continued*)

(*Continued*)

Code	Category	Description
**Bleeding**		
578.1	GI	Melena
578.9	GI	Gastrointestinal hemorrhage, NOS
626.2	Menorrhagia	Excessive menstruation
784.7	Epistaxis	Epistaxis
360.43	Intraocular	Hemophthalmos
379.23	Intraocular	Vitreous hemorrhage
362.81	Intraocular	Retinal hemorrhage
599.71	Gross hematuria	Gross hematuria
459	Other bleed	Hemorrhage, NOS
568.81	Other bleed	Hemoperitoneum
423	Other bleed	Hemopericardium
784.8	Other bleed	Hemorrhage from throat
786.3	Other bleed	Hemoptysis, unspecified
786.31	Other bleed	Acute idiopathic pulmonary hemorrhage in infants
786.39	Other bleed	Other hemoptysis
719.1	Other bleed	Hemarthrosis, site unspecified
719.11	Other bleed	Hemarthrosis, shoulder region
719.12	Other bleed	Hemarthrosis, upper arm
719.13	Other bleed	Hemarthrosis, forearm
719.14	Other bleed	Hemarthrosis, h&
719.15	Other bleed	Hemarthrosis, pelvic region and thigh
719.16	Other bleed	Hemarthrosis, lower leg
719.17	Other bleed	Hemarthrosis, ankle and foot
719.18	Other bleed	Hemarthrosis, other specified sites
719.19	Other bleed	Hemarthrosis, multiple sites

Abbreviations: GI, gastrointestinal hemorrhage; ICH, intracranial hemorrhage; LE DVT, lower extremity deep vein thrombosis; NOS, not otherwise specified; PE, pulmonary embolism; w/, with; w/out, without.
[a]Codes were searched in all secondary positions (1–24).

Supplementary Table S2 ICD-9-CM and CPT codes used to identify hematopoietic stem cell transplantation (HSCT)

Code	Description
41.01	Autologous bone marrow transplant without purging.
41.04	Autologous hematopoietic stem cell transplant without purging.
41.07	Autologous hematopoietic stem cell transplant with purging.
41.09	Autologous bone marrow transplant with purging.
41.05	Allogeneic hematopoietic stem cell transplant without purging.
41.08	Allogeneic hematopoietic stem cell transplant with purging.
41.06	Cord blood stem cell transplant.
41.91	Aspiration of bone marrow from donor for transplant.
99.79	Apheresis (harvest) of stem cells.

Abbreviations: CPT, current procedural terminology; ICD-9-CM, International Classification of Disease, Ninth Revision, Clinical Modification.

Supplementary Table S3 Risk factors associated with subsequent bleeding after incident upper extremity deep vein thrombosis among treated acute myeloid leukemia patients in California, 2009 to 2014

Variables	HR	95% CI	*p*-Value
UE DVT[a]			
Yes	2.07	(1.60, 2.68)	<0.0001
No	Reference		
HSCT[a]			
Yes	0.97	(0.69, 1.37)	0.8654
No	Reference		
Gender			
Female	1.03	(0.89, 1.18)	0.7175
Male	Reference		
Race/Ethnicity			
non-Hispanic White	Reference		
African American	1.13	(0.82, 1.55)	0.4651
Hispanic	1.25	(1.06, 1.48)	0.0093
Asian/Pacific Islander	1.48	(1.21, 1.81)	0.0002
Age at diagnosis			
<50	Reference		
50–59	0.66	(0.53, 0.83)	0.0003
60–69	0.75	(0.61, 0.92)	0.0053
≥70	0.66	(0.54, 0.81)	<0.0001
Comorbidities (within 2 y prior)			
Not available	1.05	(0.74, 1.47)	0.7997
0	Reference		
1–2	1.03	(0.74, 1.43)	0.8557
≥3	1.08	(0.77, 1.51)	0.6696

Abbreviations: AML, acute myeloid leukemia; DVT, deep vein thrombosis; HSCT, hematopoietic stem cell transplantation; UE, upper extremity; VTE, venous thromboembolism.
Note: Multivariable cox proportional hazards model is stratified by AML subtype and adjusted for the competing risk of death and VTE using Fine and Gray methodology.
[a]UE DVT and HSCT are included as time-dependent covariates.

Supplementary Table S4 Risk factors associated with subsequent bleeding after incident upper extremity deep vein thrombosis among treated acute lymphoblastic leukemia patients in California, 2009–2014

Variables	HR	95% CI	*p*-Value
UE DVT[a]			
Yes	1.62	(1.02, 2.57)	0.0393
No	Reference		
HSCT[a]			
Yes	1.18	(0.70, 1.99)	0.5275
No	Reference		
Gender			
Female	1.00	(0.82, 1.23)	0.9934
Male	Reference		
Race/Ethnicity			
non-Hispanic White	Reference		
African American	1.53	(0.95, 2.46)	0.0802
Hispanic	1.47	(1.14, 1.89)	0.0028
Asian/Pacific Islander	1.70	(1.19, 2.43)	0.0033
Age at diagnosis			
<18	Reference		
18–29	2.37	(1.83, 3.07)	<0.0001
30–39	1.49	(1.02, 2.16)	0.0396
40–49	1.82	(1.23, 2.68)	0.0026
50–59	2.45	(1.79, 3.35)	<0.0001
≥60	1.62	(1.06, 2.49)	0.0254

Abbreviations: CI, confidence interval; DVT, deep vein thrombosis; HR, hazard ratio; HSCT, hematopoietic stem cell transplantation; UE, upper extremity; VTE, venous thromboembolism.
Note: Multivariable cox proportional hazards model is stratified by comorbidities and adjusted for the competing risk of death and VTE using fine and gray methodology.
[a]UE DVT and HSCT are included as time-dependent covariates.
